# Modulatory Effect of Rutin on the Antitumor Activity and Genotoxicity of Cisplatin in Tumor-Bearing Mice

**DOI:** 10.34172/apb.2021.084

**Published:** 2020-08-05

**Authors:** Rajesh Prasad, Surya Bali Prasad

**Affiliations:** Cell and Tumor Biology Laboratory, Department of Zoology, School of Life Sciences, North-Eastern Hill University, Umshing-Mawkynroh, Shillong, Meghalaya, India.

**Keywords:** Rutin, Cisplatin, Dalton’s lymphoma, Antitumor activity, Genotoxicity

## Abstract

***Purpose:*** Cisplatin is a cancer chemotherapeutic drug that has been extensively used in the treatment of a variety of cancers. However, the full usage of cisplatin is limited due to its treatment associated development of multiple side effects in the host. In the present study, the modulatory effect of rutin, a type of flavonoid, on the cisplatin mediated antitumor activity and allied genotoxicity in ascites Dalton’s lymphoma (DL)-bearing mice were investigated.

***Methods:*** The antitumor activity was determined by calculating the percent increase in the life span of mice, cell viability and scanning electron microscopy (SEM) of DL cells. Further, the modulatory effect of rutin on the cisplatin-induced genotoxic effects in the same DL-bearing mice was assessed by the analysis of micronuclei, chromosomal aberration and sperm abnormality.

***Results:*** The combination treatment of mice with rutin and cisplatin showed a considerable increase in the life span of the DL-bearing mice depicting better antitumor efficacy. SEM of these DL cells showed severe membrane deformities in DL cells such as fusion of cell membrane, membrane blebbing, cell shrinkage, membrane folding and loss in microvilli from the tumor cell surface which may lead to cell death. Cisplatin alone treatment caused an increase in the frequency of chromosomal aberrations, micronuclei and sperms abnormality. However, the combination treatment of DL-bearing mice with rutin and cisplatin comparatively reduced these genotoxic effects.

***Conclusion:*** The overall findings suggest that rutin enhances the cisplatin-mediated antitumor activity and cytotoxicity against DL cells and at the same time diminishes the genotoxic effects induced by cisplatin in the DL-bearing mice.

## Introduction

Cancer is one of the major global human health-care issues. As per the facts and figures of the American Cancer Society (2018), the global cancer burden indicates that about 1 in every 7 deaths worldwide is caused by cancer which accounts for more than HIV/AIDS, tuberculosis and malaria combined.^1^ Chemotherapy is a type of cancer treatment where one or more drugs are used for a better remedy.^2^ In chemotherapy, cisplatin or *cis*-diamminedichloroplatinum(II) has been used as a potent drug widely applied in the treatment of many cancer types such as head and neck, lung, bladder, cervical, testicular, ovarian cancer, melanoma, lymphomas, etc.^3^ However, the treatment associated generation of various toxicities and drug resistance challenge the full application and potency of cisplatin.^4,5^ The major toxicities reported for cisplatin treatment are hepatotoxicity, gastrointestinal toxicity, nephrotoxicity, ototoxicity, and myelosuppression.^4^ It has also been reported that treatment with cisplatin develops chromosomal anomalies and micronuclei in bone marrow cells of mice^6^ and also causes testicular damage by decreasing sperm count and motility and increasing the production of abnormal sperms.^7,8^ To minimize cisplatin-mediated toxicities and resistance, several combination treatments have been used, wherein two or more drugs/agents with different pharmacological properties are often used.^9-11^

Flavonoids or bioflavonoids are a category of secondary metabolites of plants and fungal origin. Flavonoids possess a wide range of important biological and pharmacological activities which could be very helpful in promoting human health.^12^ Rutin (3, 3ʹ, 4ʹ, 5, 7-pentahydroxyflavone-3-rhamnoglucoside) is a flavonol category of flavonoid. Chemically, rutin is a glycoside combining the flavonol quercetin with the disaccharide rutinose (rhamnose and glucose). The term ‘rutin’ is derived from the plant *Ruta graveolens,* which contains rutin as its one of the main chemical constituents. Various beneficial biological properties of rutin have been reported which include anticancer, antioxidant, antidiabetic, anti-inflammatory, antibacterial, antifungal, neuroprotective, cardioprotective, hepatoprotective, nephroprotective, hepatoprotective, antiarthritis, anthelmintic and testicular protection.^13^ The antigenotoxic effects of rutin against gamma radiation and genotoxic effects induced by methotrexate in Swiss albino mice showed that rutin reduced the formation of micronuclei and chromosomal abnormalities in bone marrow cells in comparison to methotrexate group.^14,15^

Thus, considering the antitumor activity and several toxicities related to cisplatin treatment and various beneficial biological properties of rutin, the current study was undertaken to examine the modulatory effect of rutin on the cisplatin-mediated antitumor activity and genotoxicity in ascites Dalton’s lymphoma (DL)-bearing mice.

## Materials and Methods

### Chemicals

Rutin (CAS No. 153-18-4, ≥94% purity) was obtained from Sigma Co., USA and cisplatin solution was purchased from Biochem Pharmaceutical Industries, Mumbai, India. All other chemicals of analytical grade were purchased from HiMedia and SRL Pvt. Ltd., Mumbai, India.

### Maintenance of animals and tumor

The inbred Swiss albino mice colony is being maintained in typical laboratory conditions with food pellets and drinking water supplied *ad libitum*. As per the established procedure in our laboratory, the ascites DL tumor was maintained *in vivo* by serial intraperitoneal (i.p.) transplantation of viable tumor cells (1×10^7^cells in 0.25 ml phosphate-buffered saline (PBS, pH 7.4) in 11-12 weeks old mice of both sexes, After tumor transplantation, from 3-4 days onwards an elevation in the abdomen size and body weight with the slow movement of mice was observed which was an early sign of tumor growth/progression.^16^ The DL-transplanted mice generally survive for 19-20 days.

### Drugs treatment and antitumor study

The dosage and the schedule of treatment of the drugs were based on our previous reports.^17,18^ The DL-transplanted mice were randomly divided into four groups keeping ten mice per group as described below:

- Group I: mice received drug vehicle (i.e. PBS) only (DL-bearing control).
- Group II: mice were treated with rutin (30 mg/kg body weight, i.p.) on the 8^th^ and 10^th^-day of tumor transplantation.
- Group III: mice were treated with cisplatin (8 mg/kg body weight, i.p.) on the 10^th^ day after tumor transplantation.
- Group IV: mice were treated with rutin on 8^th^ and 10^th^ day followed by a single dosage of cisplatin on the 10^th^ day keeping a gap of 6-hour post rutin treatment.

The survival patterns of the mice in different groups were determined by recording daily the deaths if any, of the mice. The percent increase in life span (%ILS) and mean survival time (MST) of the DL-bearing mice under different groups were calculated to determine the antitumor efficacy of the drugs using the following formula:

$$Increase\;in\;life\;span\left(ILS\right)=\left[\frac{MST\;of\;mice\;in\;treated\;group-MST\;of\;mice\;in\;control\;group}{MST\;of\;mice\;in\;control\;group}\right]\times 100$$

$$where\;MST=\frac{\sum survival\;time\;\left(days\right)\;ofeach\;mouse\;in\;a\;group}{Total\;number\;of\;mice}$$

### Cell viability assay

The trypan blue exclusion test was performed to check the cell viability of DL cells and splenocytes.^19^ Briefly, single cell suspension of splenocytes was prepared in PBS from the mice in different groups. DL cells and splenocytes collected at different duration (i.e. 24, 48 and 96 hours) from DL-bearing mice in different groups were washed two times with PBS. Then, the fraction of the cell suspension was mixed with an equivalent volume of trypan blue dye (0.4% in PBS) and kept for 2 minutes. Viable (unstained cells) and non-viable/dead (stained cells) were counted with a Neubauer hemocytometer under a light microscope (Meiji). The dead cells in each group were calculated by monitoring 10-15 different view-fields.

$$\%\;Dead\;cells=\frac{Number\;of\;dead\;cells}{Number\;of\;viable\;cells+Numberof\;dead\;cells}\times 100$$

### Scanning electron microscopy (SEM) of DL cells

At different time intervals (i.e. 24, 48 and 96 hours), the DL cells were collected from mice in different groups and processed for SEM study. The collected DL cells were centrifuged at 1000 ×g for 10 minutes at 4°C. Further, the cell pellet was re-suspended in PBS (1:4, w/v), fixed in 2.5% (v/v) glutaraldehyde at 4°C and processed for SEM study under a scanning electron microscope (JEOL JSM-6360, SEM).

### Chromosomal aberration assay

Chromosomal aberration analysis was done as performed by Ansari et al.^20^ Briefly, 2 hours before sacrificing, the mice were treated with colchicine (2 mg/kg). Then, mice were sacrificed, and femurs were dissected out. Next, the bone marrow was flushed out with KCl (0.075 M) with the help of an insulin syringe and re-suspended in KCl (hypotonic treatment) and kept in a water bath (37°C) for 20 minutes. After hypotonic treatment, cell pellets were re-suspended in KCl and subsequently fixed in freshly prepared Carnoy’s fixative for 20 minutes and the steps were repeated 2-3 times for proper washing. The cells’ suspension was then dropped from a substantial height onto a pre-chilled clean slide and left to air dry. 5% Giemsa stain was used to stain the prepared slides and again kept to air dry. Fifty well-spread metaphase spreads per slide were thoroughly studied under a light microscope (Leitz) and different metaphase chromosomal aberrations were scored.

### Bone marrow micronuclei analysis

The micronuclei test was performed in different groups as described by Schmid (1976).^21^ Briefly, femurs of mice in different groups were separated and bone marrow was flushed out in PBS (pH 7.4) in a centrifuge tube. After centrifugation (1000 rpm, 4°C for 5 minutes), the bone marrow cells pellet was treated with a weak hypotonic solution (0.075 M KCl/saline, 1:9, v/v) and cells were fixed in Carnoy’s fixative. A drop of this suspension was smoothly smeared onto a wet chilled slide, air-dried, and stained with Giemsa stain. From each treatment group, 1000 bone marrow cells were examined under the light microscope (Leitz) for the determination of the frequency of micronuclei in the cells.

### Sperm abnormalities analysis

The sperm abnormalities analysis in the mice of different groups was performed as described by Wyrobek and Bruce.^22^ The male mice were sacrificed and the cauda epididymis was removed, minced into pieces, and kept for 20 minutes undisturbed. The spermatozoa were spread on a clean slide, semi-air-dried, and then stained with 1% aqueous eosin-Y. For the sperm abnormalities analysis in terms of head and tail shapes, five hundred sperms from each group of mice were examined.

### Statistical analysis

All the results obtained in the study were expressed as the mean ± standard deviation (SD). Statistical significance between two groups was determined by one-way analysis of variance (ANOVA) followed by a post hoc (Tukey test) to analyze the difference among multiple groups. The *P* value with ≤ 0.05 was considered statistically significant.

## Results and Discussion

### Survival patterns

**Figure 1:**
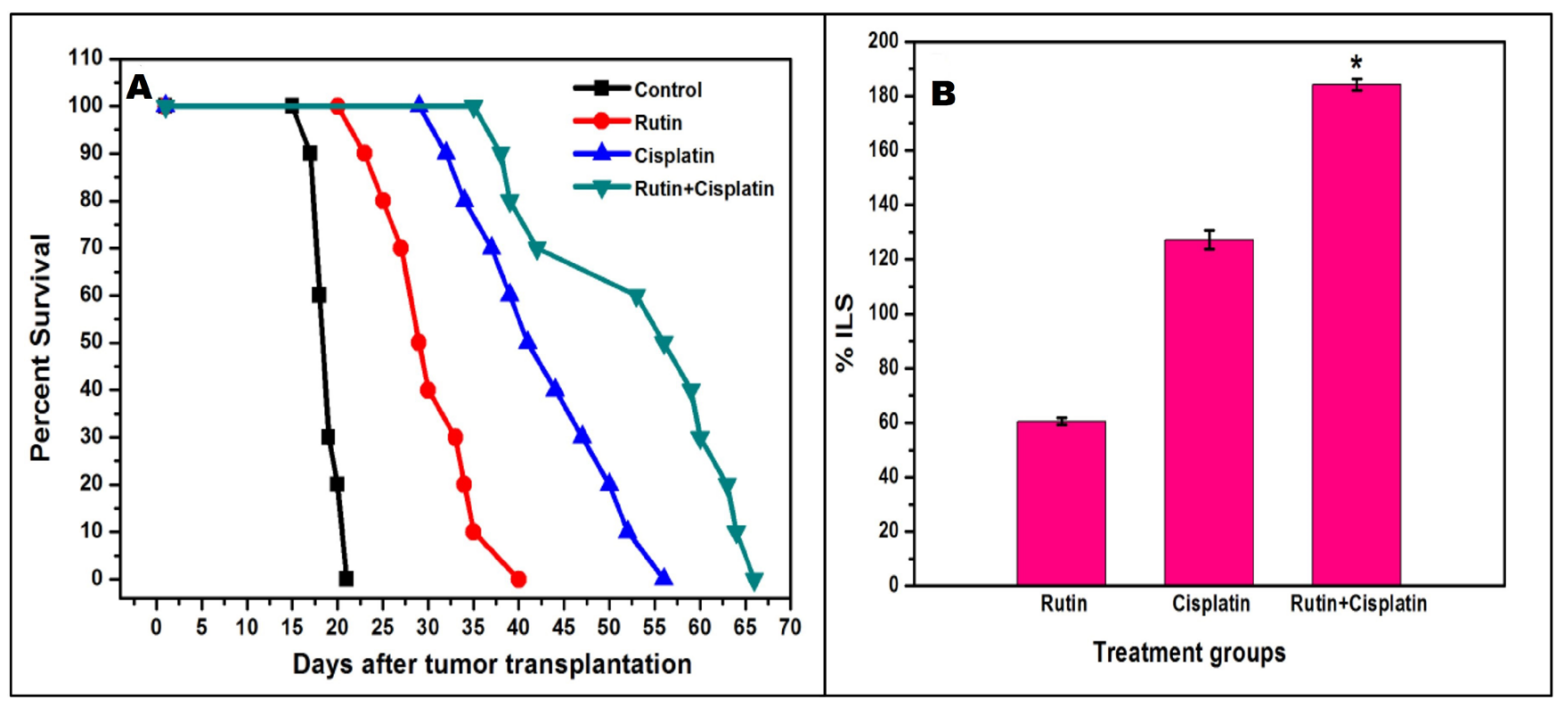
Kaplan-Meier survival curve (A), and the average percentage increase in the life span (%ILS) (B) of DL-bearing mice in different treatment groups. Results are expressed as mean ± SD of three independent experimental sets. Control= untreated tumor-bearing mice; *P≤ 0.05 as compared to cisplatin alone treatment.

It is well-known that many compounds from natural plants have chemo-preventative and chemotherapeutic efficacy against cancers. Rutin is a nontoxic and nonoxidizable bioflavonoid and possesses an antioxidant property due to which it is quite beneficial over other flavonoids.^23^ Cisplatin is useful against various cancers and in cancer therapy combination of different drugs/agents helps in reducing side effects in the host and that it is more effective compared to alone drug treatment.^24^ Murine ascites DL is a common experimental malignant tumor that has been used to evaluate the anti-cancer activity of different drugs such as cantharidin, cyclophosphamide, chlorambucil and cisplatin.^25,26^ The increase in the life span (ILS) of the treated DL-bearing mice has been considered as a reliable criterion for judging the antitumor efficacy.^25,27^ The findings on the survival patterns and ILS of DL-bearing mice showed that the MST of mice treated with rutin and cisplatin alone was about 30.5 days (ILS ~ 60.53%) and 43.2 days (ILS ∼ 127.27 %) respectively. However, the MST of mice treated with a combination of rutin and cisplatin considerably enhanced to about 54 days(ILS ∼ 184.21 %) (Figure 1). Thus, the results of the hosts’ survival patterns in different groups may suggest that combination treatment with rutin plus cisplatin against murine ascites DL has a superior beneficial approach as compared to the alone treatment. Earlier it has been revealed that rutin induces apoptosis and decreases antioxidant ability (glutathione level) in DL cells.^18^ Also, the molecular interactions of rutin with antiapoptotic proteins, Bcl-xL, c-FLIP, and glutathione-related enzymes (glutathione S-transferase and glutathione reductase) showed substantial binding energy indicating the potency of inhibition of these proteins/enzymes.^18^ The increase in apoptosis along with a decrease in glutathione in DL cells could be an important segment in the increasing life span of these mice. Further, in different murine tumor models study has also shown that rutin improves the survival time of tumor-bearing mice.^28,29^ It has been reported that administration of rutin (20 mg/kg) to nude mice bearing SW480 tumor exerted effective antitumor effect with a 156% increase in life span.^29^

### Cells viability of DL cells and splenocytes

**Figure 2:**
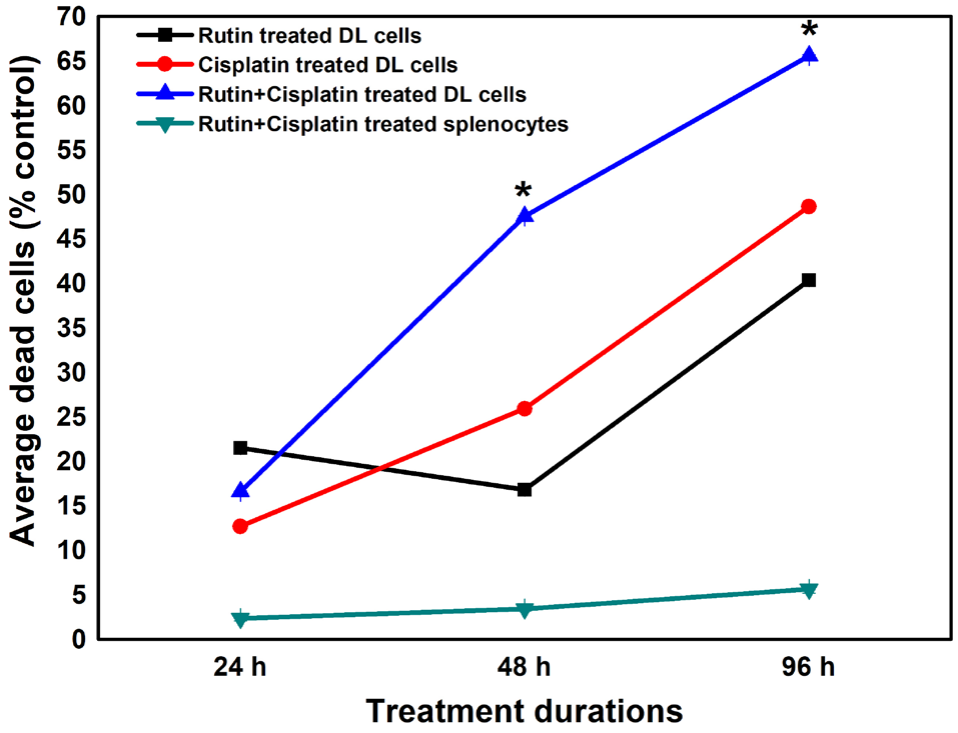
Pattern of cell deaths in DL cells and spleen cells from mice under different treatment

The viability analysis of DL cells and splenocytes revealed that the treatments caused more death in DL cells as compared to splenocytes in a time-dependent manner (Figure 2). The highest DL cells death was noted in the combination treatment at 96 hours compared to the alone treatment and at the same time, the combination treatment was less cytotoxic/sensitive to the normal splenocytes as compared to DL cells (Figure 2). The cytotoxic effects of rutin have also been studied on numerous other cancer cell lines.^29-31^ The *in vitro* antiproliferative/cytotoxic activity of rutin and orlistat has also reported inducing caspase 3/7 activity and apoptosis on the human breast cancer (MCF-7) and pancreatic cancer (PANC-1) cell lines.^31^ In another study, rutin treatment had shown significant cytotoxic effects on the proliferation of mouse leukemiaWEHI-3 cell line as well as inhibition intraperitoneally injected WEHI-3 cells in BALB/c mice *in vivo*.^32^ The present findings corroborate these reports on the cytotoxic effect of rutin and further propose that the combination of rutin and cisplatin exerts a better cytotoxic effect on DL cells.

conditions. Results are expressed as mean ± SD of three independent experimental sets. *P ≤ 0.05 as compared to cisplatin alone treatment.

### SEM of DL cells

**Figure 3:**
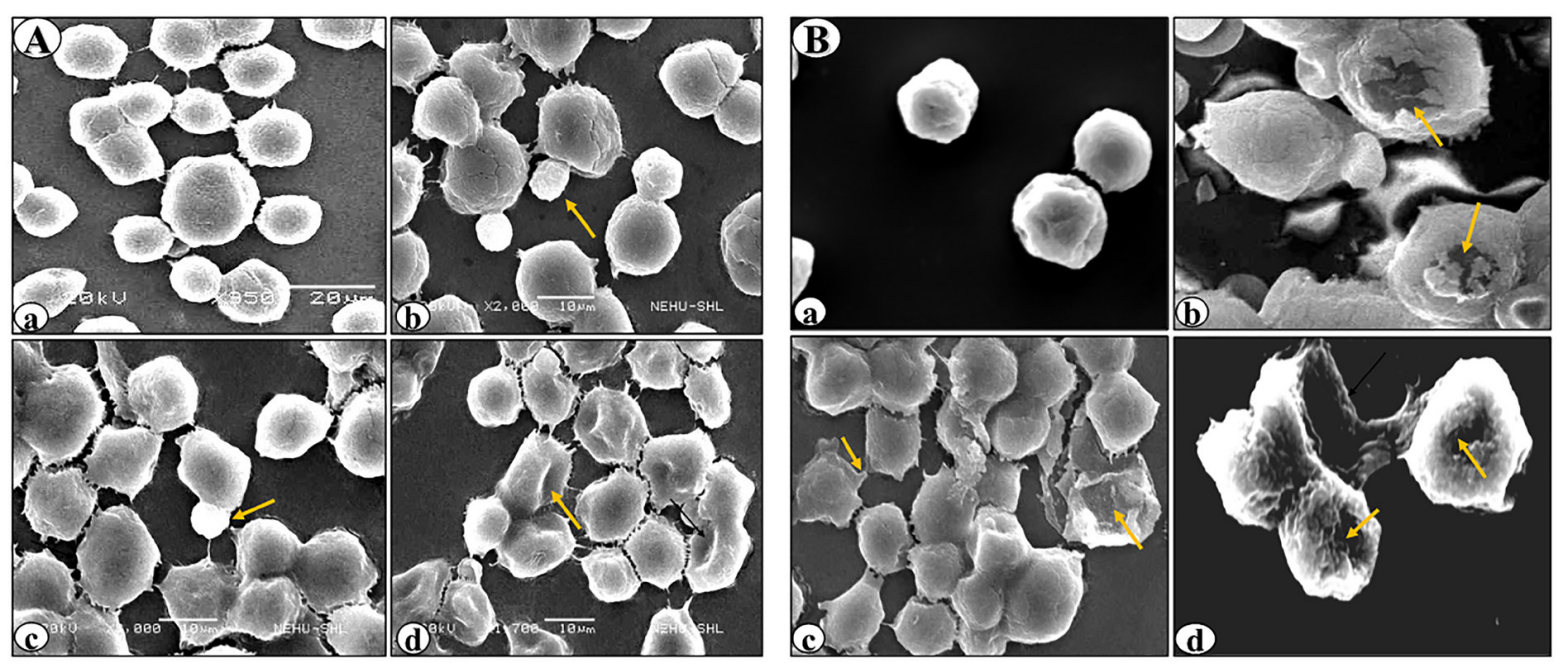
Scanning electron micrographs of DL cells treated with rutin (A) or cisplatin alone (B). (a), (b), (c) and (d) denote control, 24h, 48h and 96h of the treatment. Arrows indicate anomalous cellular features on the cells such as membrane blebs, cell membrane fusion, and cell membrane deformities.

**Figure 4:**
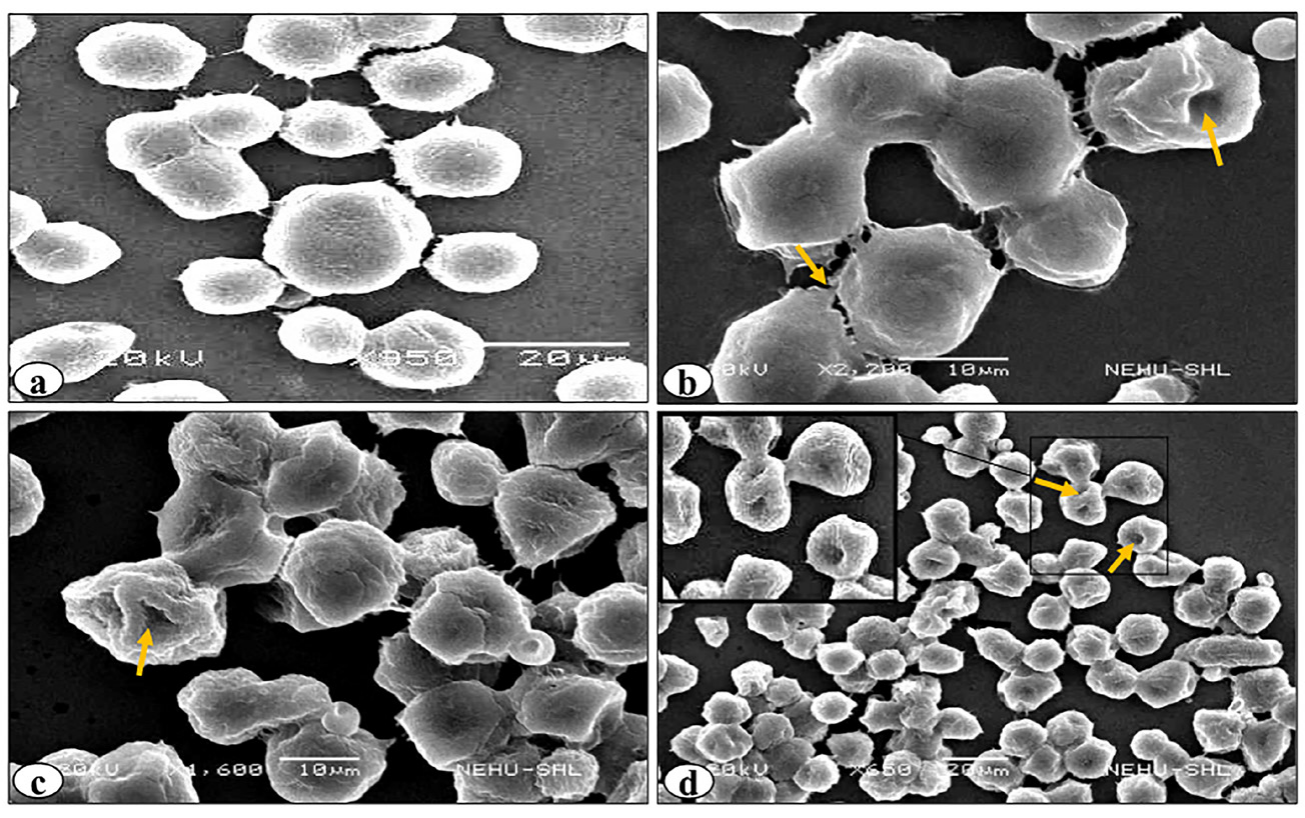
Scanning electron micrographs of DL cells treated with rutin and cisplatin combination. (a), (b), (c) and (d) denote control, 24h, 48h and 96h of the treatment respectively. Arrows indicate abnormal cellular features on the cells such as membrane blebs, cell membrane fusion, and cell membrane deformities.

SEM revealed various surface membrane deformities in DL cells under different treatment conditions (Figures 3 and 4). Uniformly scattered ruffles and membrane projections were observed on the control DL cells. After 24-96 hours of rutin and cisplatin alone treatment, some membrane blebbing, cell membrane fusion, and deformities were noted on surfaces of DL cells (Figure 3). However, rutin plus cisplatin combination treatment caused severe membrane deformities in DL cells such as fusion of plasma membrane, membrane blebbing, cell shrinkage, membrane folding, and loss in microvilli on the cell surface as compared to alone treatment (Figure 4). As observed in viability analysis, the viability of DL cells decreased more prominently in combination treatment (Figure 2), the SEM observations of DL cells support the findings on the cell viability and host survivability that combination treatment could be more effective against DL cells as compared to their alone treatment.

### Analysis of induction of bone marrow micronuclei formation, chromosomal aberration and sperm abnormality

**Figure 5:**
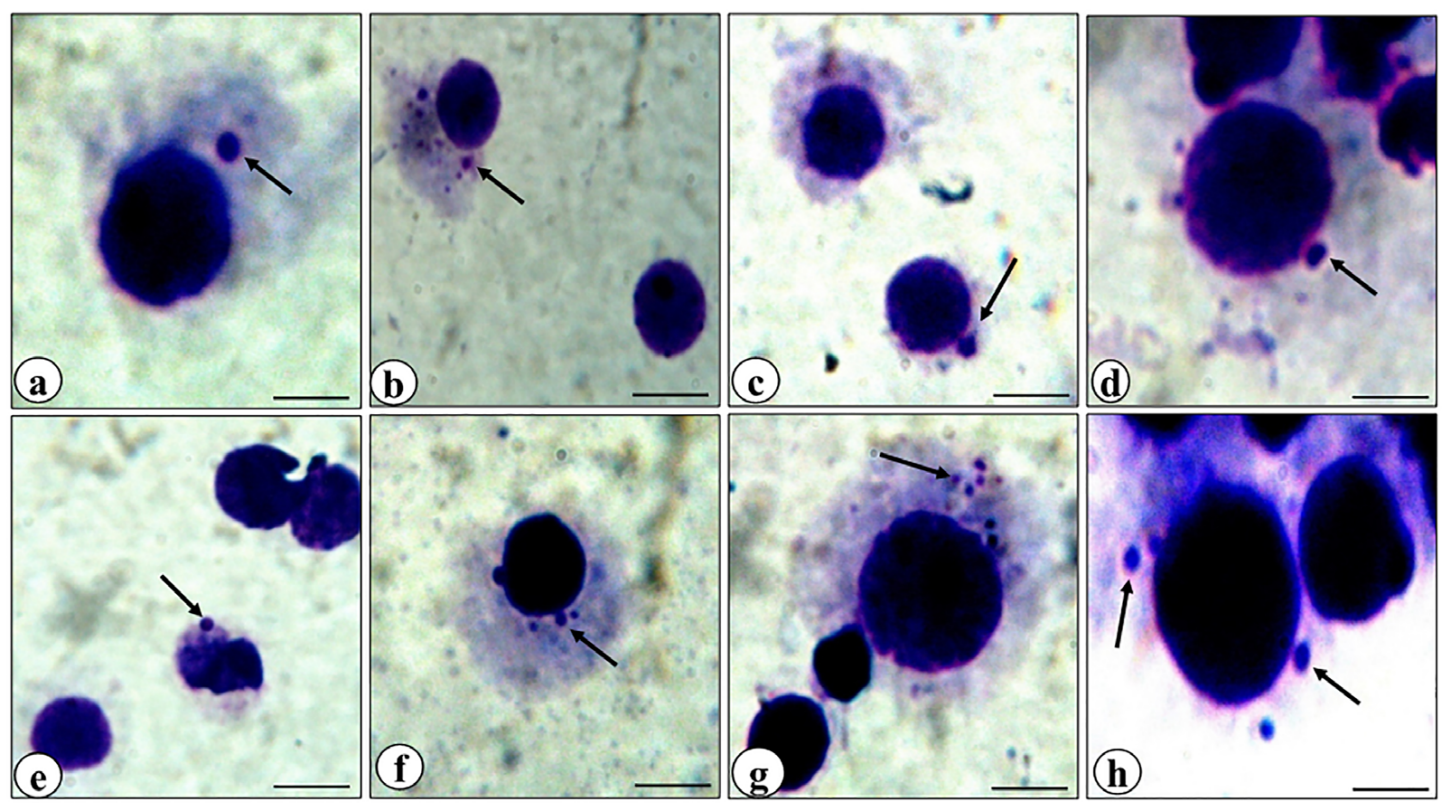
Representative photomicrographs of bone marrow cells of mice showing micronuclei (arrows). Control (a), rutin alone treatment (b, c), rutin plus cisplatin combination treatment (d, e), cisplatin alone treatment (f, g, h).

**Figure 6:**
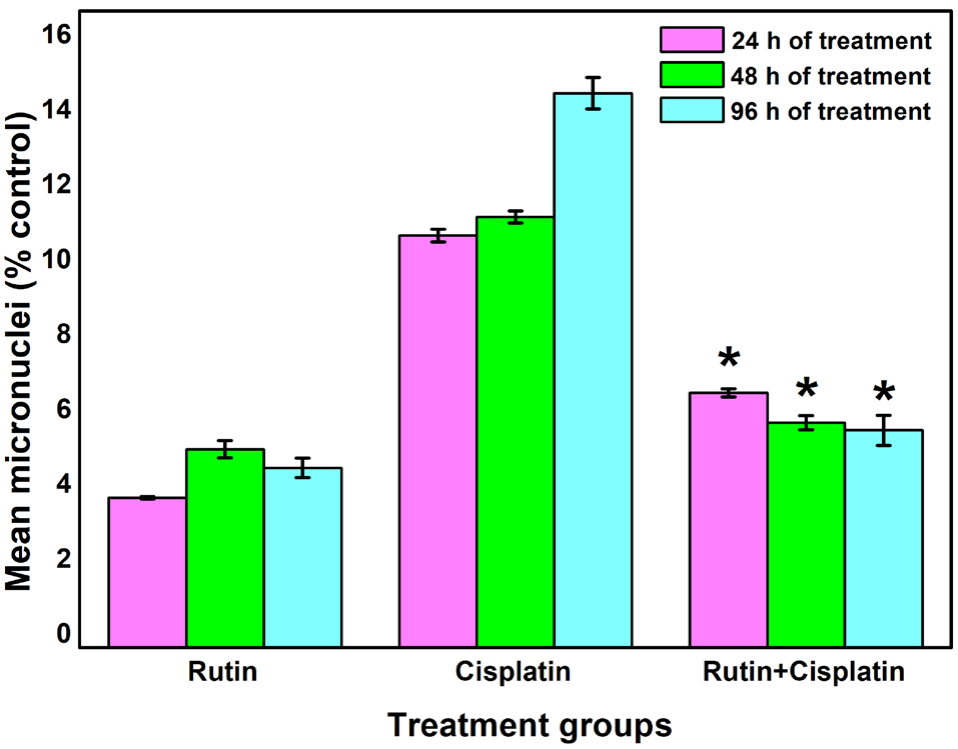
Changes in mean micronuclei development in bone marrow cells under different treatment conditions. Results are expressed as mean ± SD, n = 3; *P≤ 0.05 as compared to cisplatin alone treatment.

In the mutagenic/genotoxic bioassays of drugs, one of the best biological indicators includes induction of chromosomal aberrations, micronucleus, and sperm abnormality.^9,33^ The micronuclei are formed when a fragment of a chromosome is not integrated into one of the daughter cells during the anaphase of the cell division. The results of the micronuclei assay showed that cisplatin alone treatment group significantly increased the occurrence of micronuclei compared to the other treated groups (Figures 5 and 6). Rutin alone treatment did not significantly induce micronuclei formation. However, the development of micronuclei was considerably reduced in the combination treatment of rutin plus cisplatin as compared to cisplatin alone treatment (Figure 6). Rutin, having antioxidant properties has been reported to exhibit protective effects against 2,5-hexanedione-induced DNA and oxidative damages in rats.^34^ Further, the antigenotoxic potential of rutin and quercetin in Swiss mice exposed to gamma radiation has also been observed.^14^

**Figure 7:**
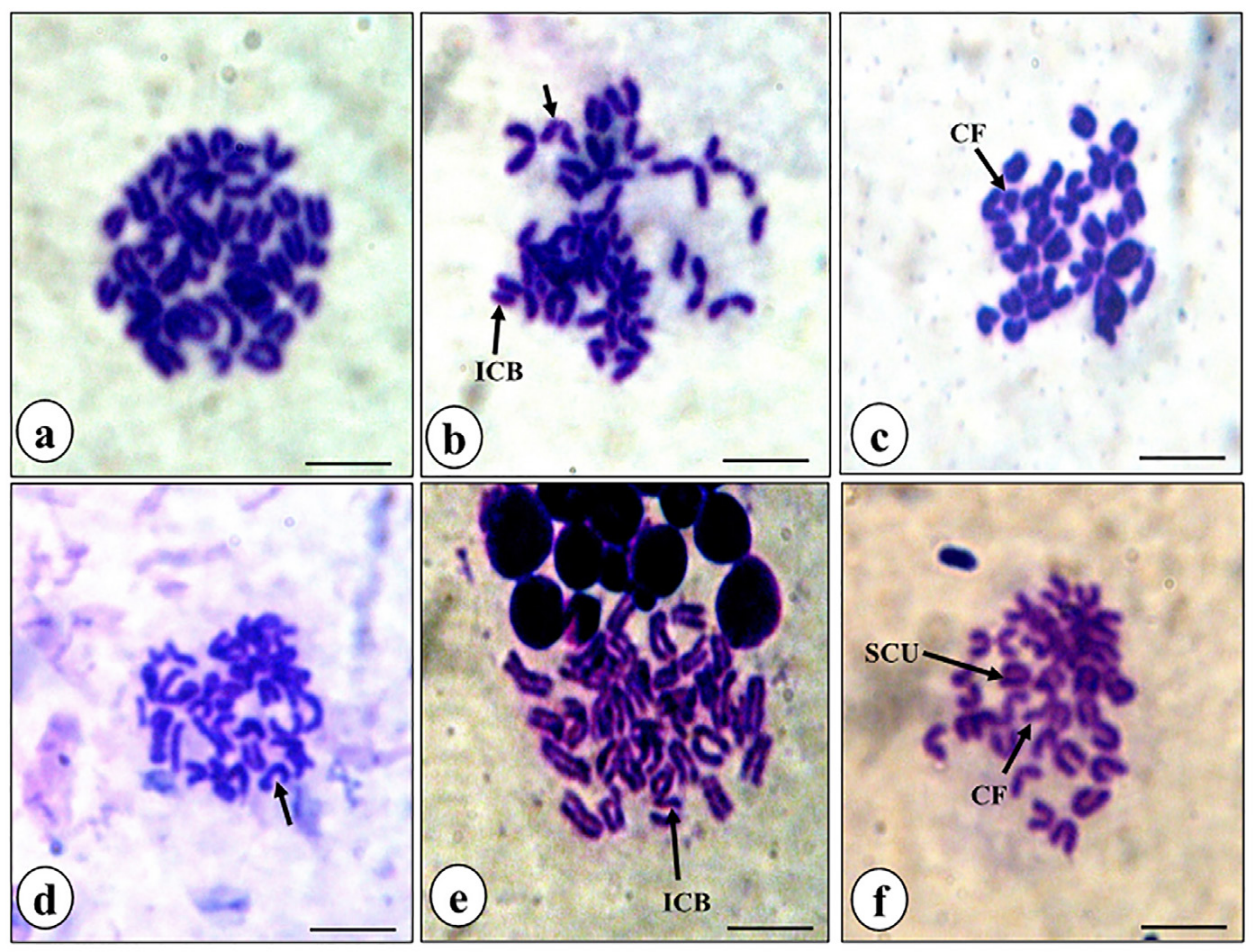
Representative photomicrographs of bone marrow metaphase chromosome spreads. Control, showing normal set of chromosomes (a), rutin alone treatment (b), rutin plus cisplatin combination treatmen (c), and cisplatin alone treatment((d-f). Different types of common aberrations (arrows) observed were isochromatid breaks (ICB), chromosomal fragments (CF) and sister chromatid unions (SCU).

**Figure 8:**
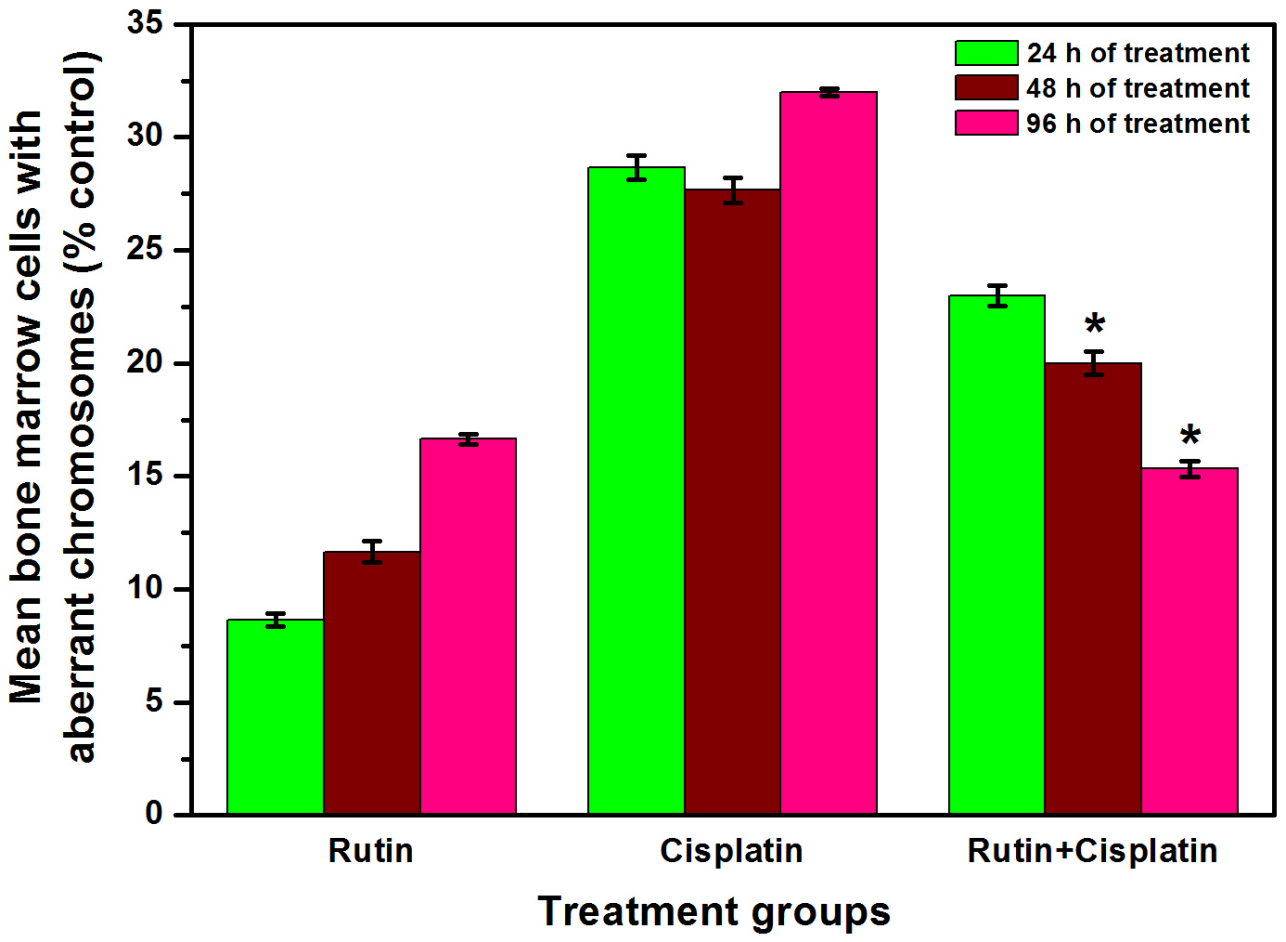
Percent bone marrow cells showing mean aberrant metaphases in different groups. Results are expressed as mean ± SD, n = 3; *P≤ 0.05 as compared to cisplatin alone treatment.

It is well-known that cisplatin treatment causes various chromosomal aberrations such as structural aberrations (deletion, fragmentation, centric fusion, ring, break, and gaps) and numerical aberration (monosomy and trisomy).^35^ In the present study also, cisplatin treatment caused a considerable augmentation in the aberrant metaphase chromosomes in the bone marrow cells of DL-bearing mice (Figures 7 and 8). However, a significant reduction in mean aberrant chromosomes was observed in combination treatment as compared to cisplatin alone treatment (Figure 8). In a study on bone marrow cells of Swiss albino mice, rutin in combination with methotrexate was reported to ameliorate the methotrexate-induced micronuclei formation and chromosomal aberrations in the cells.^15^ In a different study, rutin obtained from the extract of *Olea europaea* L. leaves was reported to show antimutagenic activity in mouse bone marrow after X-ray irradiation.^36^ It has also been demonstrated that 2,5-hexanedione- induced oxidative and DNA damages in the viscera of Wistar rats was ameliorated by rutin administration *ex vivo*.^37^

**Figure 9:**
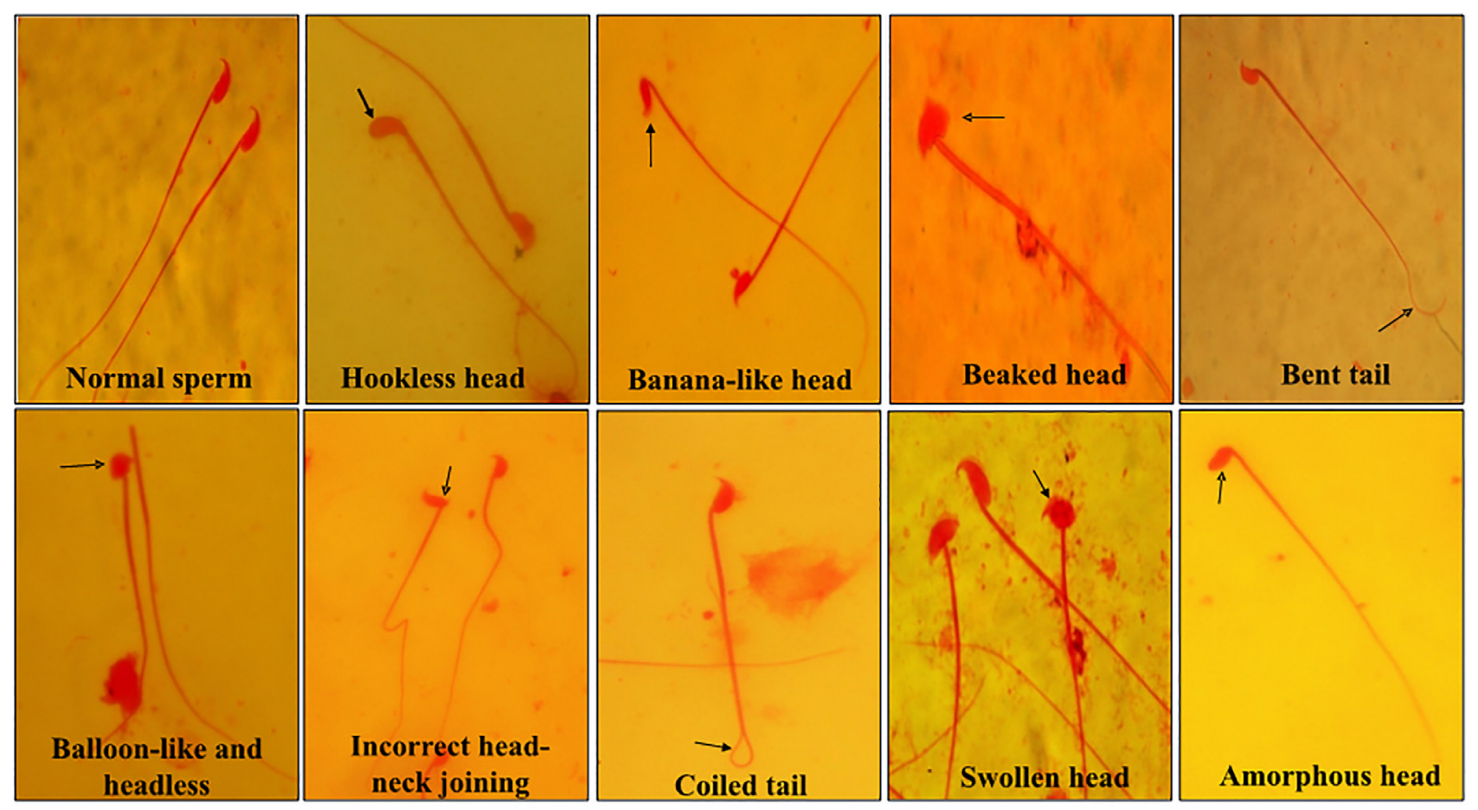
Representative photographs of spermatozoa from mice showing various types of abnormalities under different treatment conditions such as hookless head, banana-like head, beaked head, bent tail, incorrect head-neck joining, balloon-like head, swollen head, amorphous head, coiled tail and headless.

**Figure 10:**
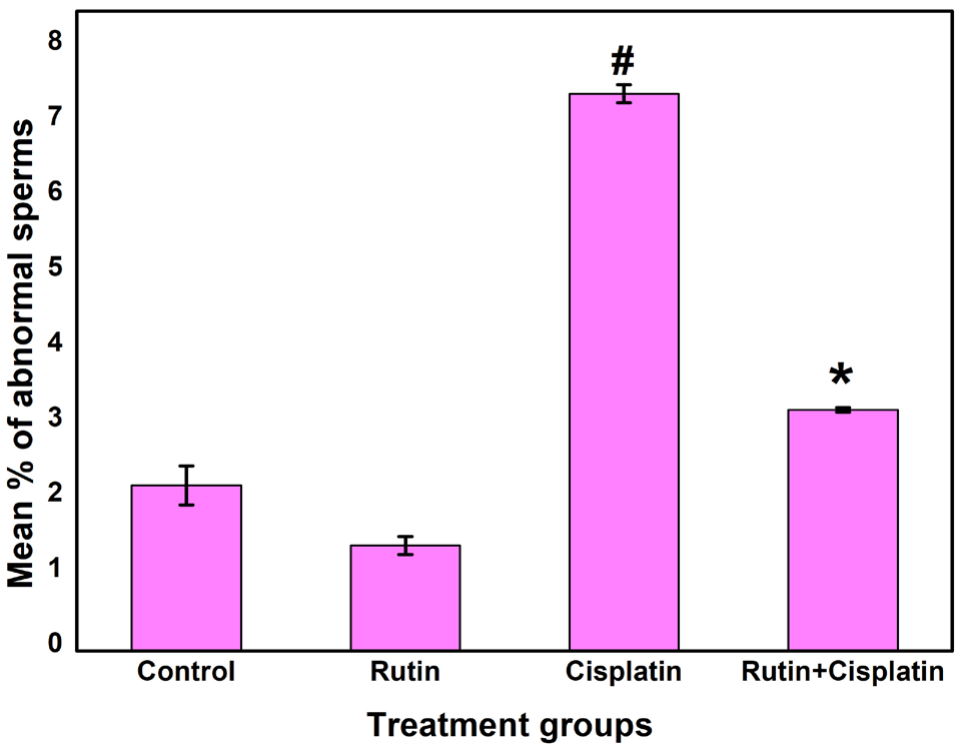
Sperms abnormality variation in mice in different groups under different treatment conditions. Results are expressed as mean ± SD, n = 3; *P≤ 0.05 as compared to cisplatin alone treatment and #*P*≤ 0.05 as compared to the control.

It has been reported that cisplatin treatment causes testicular/reproductive organ toxicities in the male. Cisplatin treatment to rats develops testicular toxicity and some of the reasons for this toxicity induction are the increase in the number of sperms with abnormal morphology, chromosomal abnormalities in spermatozoa, a decline in reproductive organ weights and decrease in number and motility of spermatozoa.^38,39^ The results of sperm abnormality analysis revealed that cisplatin alone treatment exhibited a substantial increase in sperm abnormalities as compared to other treated groups (Figures 9 and 10). The abnormalities observed in the morphology of head and tail of sperms were as hookless head, banana-like head, beaked head, bent tail, incorrect head-neck joining, balloon-like head, swollen head, amorphous head and coiled tail and headless (Figure 9). The mice treated with the combination of rutin and cisplatin showed significantly reduced frequency of sperm abnormalities compared to the cisplatin alone treatment (Figure 10). These findings are in good agreement with the earlier studies.^40,41^ It has been reported that rutin in combination with cisplatin treatment lowers the sperm abnormality compared to cisplatin alone group in adult male rats.^42^ In another study, it was demonstrated that cisplatin alone treatment resulted in a significant decrease in daily sperm production, a decrease in head length and percent DNA in head and reduction of the number of spermatogonia, spermatocytes and spermatids. However, rutin co-treatment resulted in reversing these effects and ameliorated the cisplatin-induced reproductive toxicity in male rats.^39^

Our findings demonstrate the modulatory role of rutin against genotoxicity induced by cisplatin in the host.

## Conclusion

Overall findings from the present study suggest that rutin augments the cisplatin-mediated antitumor activity and cytotoxicity against DL cells and simultaneously assists in lessening the occurrence of chromosomal aberrations, micronuclei and sperm abnormality in DL-bearing mice induced by cisplatin.

## Ethical Issues

The maintenance, use of the mice and the experimental protocols have been approved by the Institutional Animal Ethics Committee (No. NEC/IEC/2018/005, dated October 01, 2018) of the North-Eastern Hill University, Shillong, India.

## Conflict of Interest

The authors declare that there is no conflict of interest in the present study.

## Acknowledgments

The authors gratefully acknowledge the Department of Zoology, North-Eastern Hill University, Shillong for providing the required research facilities. We thank Grimchi T. Sangma for helping in the correction of the manuscript.
